# Grading evaluation of haploid fertility restoration traits based on inception-ResNet in maize

**DOI:** 10.1016/j.plaphe.2025.100140

**Published:** 2025-11-07

**Authors:** Yizheng Wang, Zhou Yao, Wenhao Song, Kai Jiao, Fankun Zeng, Junli Deng, Yingjie Xiao, Zuxin Zhang, Jianbing Yan, Jianxiao Liu, Yanzhi Qu

**Affiliations:** a National Key Laboratory of Crop Genetic Improvement, Huazhong Agricultural University, Wuhan, 430070, China; b Hubei Hongshan Laboratory, Wuhan, 430070, China; c College of Informatics, Huazhong Agricultural University, Wuhan, 430070, China; d College of Plant Science, Huazhong Agricultural University, Wuhan, 430070, China; e Yazhouwan National Laboratory, Sanya, 572024, China

**Keywords:** Maize, Haploid, Deep learning, Seed setting rate, Anther emergence rate

## Abstract

Double haploid (DH) technology can significantly shorten the breeding cycle and improve the breeding efficiency, and it is favored by breeders. The metrics for evaluating the effect of haploid genome doubling mainly include anther emergence and ear seed setting. The evaluation of fertility restoration ability is mainly conducted through visual inspection at present, which is time-consuming, and easy to be affected by fatigue, resulting in errors and inconsistencies. Therefore, it is urgent to develop efficient and accurate evaluation technology to reduce the field work burden of researchers. In this work, we propose a grading evaluation model (Maize-IRNet) of haploid anther emergence and ear seed setting based on Inception-ResNet. Firstly, the modules of Stem and Inception-ResNet are utilized for image feature extraction and multi-scale feature learning. Then, the Reduction module is used for spatial downsampling and feature compression, and the global attention mechanism (GAM) is used to enhance the recognition of key regions of the image. The experimental results show that the Maize-IRNet's classification accuracy of haploid ear seed setting and anther emergence is 84.2 ​% and 84.0 ​%, which is higher than six baseline methods (VGG11_bn, ResNet50, ResNet101, ViT-Base-16, gMLP, MLP-Mixer). In order to facilitate the practical application for breeding researchers, we have developed a mobile application that integrates the Maize-IRNet model. This study helps to achieve high-throughput collection of fertility restoration phenotypes, improves the evaluation efficiency of fertility restoration, reduces breeding costs, and provides technical support for the promotion of engineering breeding of DH technology.

## 1 Introduction

As an important food and feed crop, maize has a direct impact on the agricultural economy and industrial production [1]. The rate of production increase of major crops in the future is not enough to meet the demand for food [2]. Therefore, rapid breeding of new high-yielding and high-quality crop varieties is critical for dealing with the challenges of global food security. The traditional inbred line breeding needs 6–8 generations of continuous selfing, but the DH technology only needs 2–3 generations to select homozygous inbred lines, which significantly accelerates the breeding process. In recent years, DH technology has achieved theoretical and technical breakthroughs in vivo haploid induction and haploid sorting. Researchers have cloned several haploid generation related genes and elucidated the haploid generation mechanism [[3], [4], [5], [6]]. Haploid sorting technology realizes the expansion of sorting objects from seed stage to immature embryo stage, and achieves high-throughput and automatic identification of haploid seeds [7,8]. However, the theoretical and technical research of haploid doubling has not achieved a breakthrough. The development of high-throughput phenotyping and artificial intelligence technologies facilitates the engineering application of DH technology, thereby accelerating the breeding process and enhancing breeding efficiency.

The chromosome doubling efficiency and fertility restoration are commonly used to assess the status of haploid chromosome doubling. Chromosome doubling efficiency is used to evaluate the effects of different reagents, treatment durations, and methods on chromosome doubling. Fertility restoration is mainly used to measure the extent of spontaneous doubling in haploids without chemical treatment. The variation range of haploid fertility restoration across inbred lines with different genetic backgrounds is 0.61–77.6 ​% [9]. Several factors contribute to this variation, including genetic background, cytological mechanisms, and environmental conditions. The genetic background represents the QTLs and their number associated with spontaneous haploid genome doubling (SHGD) carried by specific materials. The responsiveness of different materials to the environment can also lead to distinct levels of haploid fertility restoration. At present, researchers mainly use linkage analysis, association analysis, and segregation distortion analysis to conduct QTL mapping studies of SHGD. To date, more than 50 QTLs associated with SHGD have been located worldwide. However, most studies remain at the preliminary mapping stage. For example, Ren et al. [10,11] identified several QTLs (e.g., *qhmf4*, *qshgd1*) through segregation distortion analysis using materials with high spontaneous doubling. Ma et al. [12] detected 14 SNPs associated with fertility restoration through GWAS analysis, and further analyzed the potential roles of candidate genes in meiosis. Trampe et al. [13] and Verzegnazzi et al. [14] located some identical QTLs related to fertility recovery traits using GBS and GWAS, respectively. However, the detected QTL regions are large and exhibit low recombination frequency, and the candidate genes within these regions have not yet been identified. Therefore, the genetic basis underlying SHGD requires further investigation.

Haploid fertility restoration involves two parts: the ear and the tassel. The emergence of anthers on the tassel is a key indicator of evaluating the extent of male fertility restoration. Anther emergence directly reflects the efficiency of male gamete doubling and the ability to produce fertile pollen. The ear seed setting rate is an indicator of female gamete doubling, and it reflects the number of seeds produced after receiving fertile pollen. Together, tassel anther emergence and ear seed setting determine the overall level of haploid chromosome doubling. Therefore, accurate and efficient identification of haploid tassel anther emergence and ear seed setting is essential for evaluating the effect of haploid chromosome doubling in maize and improve the QTL mapping accuracy of SHGD. Traditional methods for investigating haploid tassel anther emergence and ear seed setting in maize rely mainly on manual evaluation. Field surveys of haploid fertility restoration traits are labor-intensive and highly dependent on the professional skills and experience of the investigators. An experienced worker typically requires 3–5 ​s to record the trait of an individual plant, allowing for the evaluation of approximately 700–1200 plants per hour. However, prolonged fieldwork and complex environmental conditions often reduce the survey speed significantly. In addition, there may be significant differences in the phenotype survey results among different staff members. Even experienced workers may make errors when judging intermediate levels of fertility restoration after long working hours. Inexperienced workers are only able to accurately identify extreme phenotypes, while their evaluation of the intermediate levels is highly uncertain. Therefore, the field phenotype investigations of haploid fertility restoration traits are often conducted by the same person to avoid inconsistent results due to different evaluation criteria.

With the development of machine learning and deep learning, image recognition technology has gradually become a critical solution to address this problem. The image processing technology based on artificial intelligence is expected to identify the tassel anther emergence and ear seed setting of maize haploid efficiently and accurately, thus improving the engineering level of DH technology. In recent years, the rapid development of deep learning and computer vision technology has provided a new technical paradigm for crop phenotype analysis. The technical system has been extended from general agricultural detection tasks (such as rice disease classification [15] and wheat variety identification [16], *etc*.) to refined researches of crop breeding. The related researches focus on improving the accuracy of image analysis in the agricultural field by combining attention mechanisms with lightweight network architectures. For example, Li et al. [17] significantly improved the robustness of identifying maize diseases in complex environments by combining the lightweight network MobileNet and Convolutional Block Attention Module (CBAM). Similarly, Zhao et al. [18] further improved the defect detection efficiency for soybean seeds by 17 ​% through fusing the modules of ShuffleNet into MobileNetV2. In addition, the multimodal data fusion strategies are becoming increasingly used in agricultural image analysis, including the framework of fusing visible light and near-infrared spectroscopy [19] and the system of integrating UAV remote sensing and 3D point cloud technology [20]. These methods promote the paradigm innovation of crop phenotype analysis from static measurement of single trait to dynamic monitoring in the whole growth cycle. In the field of crop phenotyping, deep learning-driven image processing technologies have covered from basic research to engineering applications. At present, deep learning-based image processing algorithms are widely applied in multiple aspects of crop phenotyping. The related applications include disease identification and resistance evaluation [21], organ detection and growth monitoring [22], seed quality assessment [23], and seed classification [24], *etc*. These applications are mainly categorized into YOLO-based detection models and multi-scale attention networks according to the technical approaches. In the field of object detection for crop phenotyping images, researchers have mainly developed methods of detecting specific targets in plants based on the YOLO series algorithms. For example, Light-YOLO has been used to detect cottonseed breakage [25], PCSA-YOLO to detect fusarium head blight (FHB) in wheat [26], and S-YOLO to monitor flowering stages [27]. At the same time, researchers have designed specialized neural networks to extract multi-scale features according to crop morphology and texture. For instance, researchers have used ResNeXt to count wheat spikes [28]. They have also applied MLAENet with a lightweight multi-scale attention mechanism for maize tassel counting under complex backgrounds in field [29]. The multi-scale attention map mechanism of MSANet has been adopted for soybean seed counting [30]. In addition, some researchers have integrated the developed image analysis algorithms into applications for practical field phenotyping. Liu et al. [31] deployed the Grain-YOLO model on an Android application, enabling real-time rice grain counting in actual field conditions. Bao et al. [32] implemented a deep learning network on smartphones to accurately identify citrus maturity stages. These works demonstrate that deep learning technologies are overcoming the limitations of theoretical studies, and they are now widely applied to phenotyping in real field environments.

As demonstrated above, doubled haploid breeding, genetic studies on SHGD, and the exploration of optimal chemical doubling methods are of critical importance. Precise and efficient assessment of haploid fertility restoration phenotypes will accelerate breakthroughs in research on haploid genome doubling. It can be seen that there are some studies of analyzing maize phenotype images using deep learning, but there are few researches on the prediction of haploid ear seed setting and tassel anther emergence in maize. Our work mainly includes the following aspects.

(1) Constructed an image dataset for maize haploid fertility restoration evaluation. We constructed a dataset containing 1897 high-resolution haploid ear images with different seed setting rates and 6443 tassel images with different anther emergence rates. According to the phenotypic characteristics of each image, we annotated its level to ensure the high availability of the dataset in the grading evaluation of fertility restoration traits.
(2) We developed a maize grading evaluation model (Maize-IRNet) of haploid fertility restoration trait based on Inception-ResNet. Maize-IRNet includes the modules of Stem, Inception-Resnet, Reduction and GAM. Firstly, the Stem module is used to perform preliminary feature extraction and feature dimensionality reduction. Then the Inception-ResNet module is used for multi-scale feature learning, and combined with the Reduction module to achieve feature downscaling and compression. Finally, the GAM is used to enhance the attention to the key regions of the image to improve the detection accuracy of Maize-IRNet. The experimental results show that Maize-IRNet has higher prediction accuracy than the traditional methods of VGG, ResNet, ViT, gMLP, and MLP-Mixer.
(3) Developed a user-friendly mobile application (APP). We have developed an Android mobile application of Haploid-Fertility that integrating the Maize-IRNet algorithm. Users can take and upload the ear and tassel images of maize haploid, and then obtain the grading results of seed setting rate and anther emergence rate in real time, which improves the accuracy and efficiency of field phenotype collection of haploid plants.

## 2 Materials and methods

### 2.1 Material planting and maize haploid fertility restoration image acquisition

In this study, we performed field phenotype investigations using haploid populations with different genetic backgrounds. We first generated F_1_ hybrids by crossing the maize inbred lines DX with B73, Mo17, Zheng58, and KN5585, as well as RL36 with B73 and M119. These hybrids were subjected to parthenogenetic induction using the haploid inducer line YHI-1 with a haploid induction rate greater than 10 ​% (HIR >10 ​%). Four DX-background haploid populations were planted at the southern propagation base of the Jilin Academy of Agricultural Sciences in Ledong, Hainan Province, in November 2021. In May 2023, haploid populations from the RL36 hybrid backgrounds were sown at the Zhangye research station of the Gansu Academy of Agricultural Sciences. The first batch of tassel and ear images was collected at the Hainan site between January and March 2022, totaling 2448 images. The second batch, comprising 5892 tassel images, was collected at the Gansu site in August 2023. Images were captured using a Huawei Honor 10 smartphone equipped with a 16-MP color and a 24-MP monochrome dual-camera system. Shooting distance was maintained at 20–25 ​cm with a black background placed behind the objects to ensure consistency and minimize interference. Finally, we collected a total of 8340 high-resolution images of maize haploid fertility restoration from two field sites across two growing seasons.

### 2.2 Preprocessing and grading of maize haploid fertility restoration images

**Fig. 1:**
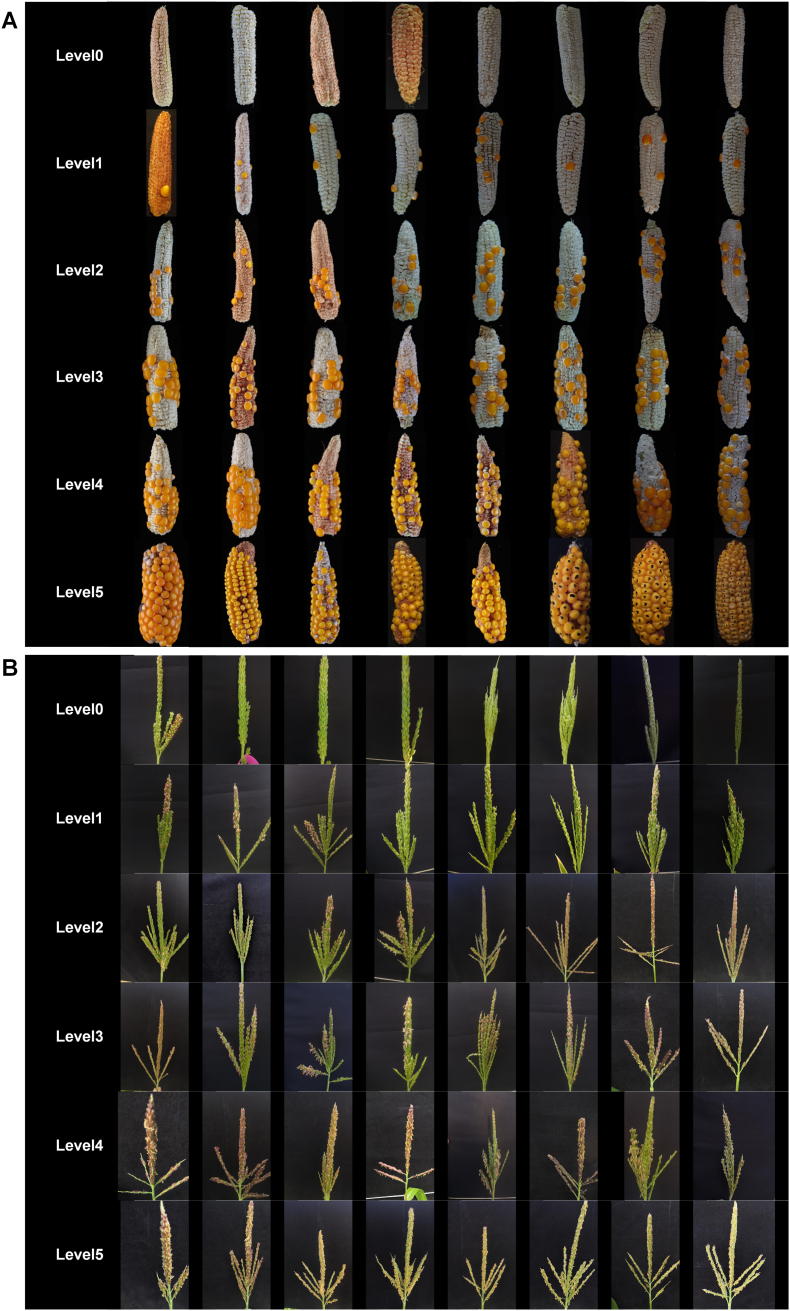
Phenotypic variation of maize haploid fertility restoration. (A) Different levels of seed setting in maize haploid ear. (B) Different levels of anther emergence in maize haploid tassel.

Firstly, we performed the following preprocessing operations on the original 8340 images, which contained 1897 ear images of haploid plants and 6443 tassel images: (1) removing blurred or low-quality images; (2) adjusting the resolution to 2048 ​× ​2048 pixels; (3) according to the reported grading standards of maize haploid fertility restoration traits [33], we divided the images into six categories based on the degree of anther emergence and seed setting. The grading criteria used for classification are elaborated in Table S1. The numbers of images in each level of tassel anther emergence and ear seed setting are shown in Table S5. The representative examples of different levels of anther emergence and seed setting are shown in Fig. 1. The sample sizes of the six levels of anther emergence are shown as follows: level 0: 3279; level 1: 1145; level 2: 500; level 3: 487; level 4: 422; level 5: 610. The sample sizes of the six levels of ear seed setting are shown as follows: level 0: 233; level 1: 498; level 2: 321; level 3: 315; level 4: 210; level 5: 320.

### 2.3 Methods

**Fig. 2:**
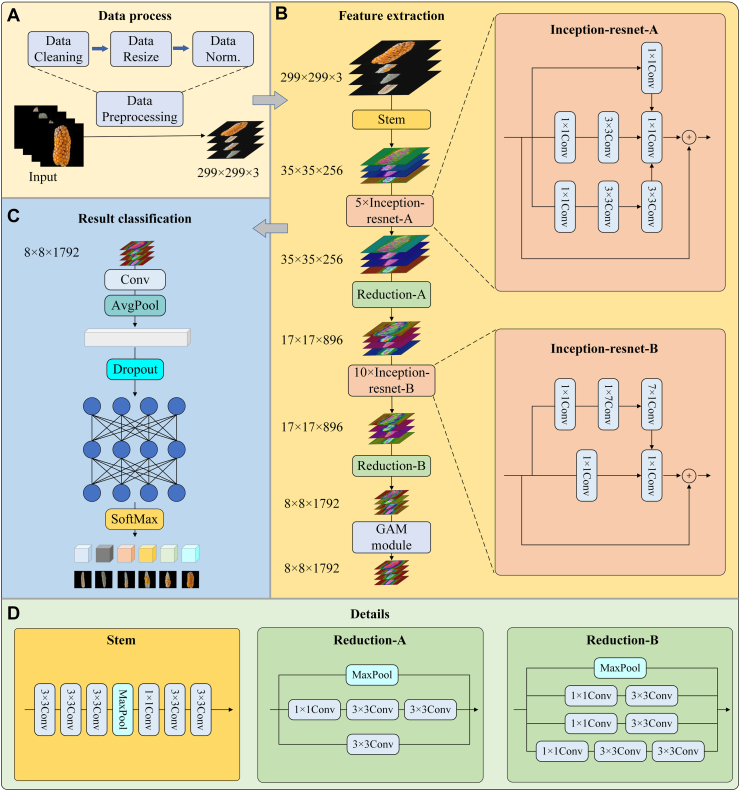
The overall structure of the Maize-IRNet network. (A) Data preprocessing pipeline. Using standard preprocessing operations of cropping and normalization to process the input images. (B) Feature extraction module. The backbone network of Maize-IRNet can extract multi-scale features of images, and integrate the global attention mechanism to strengthen the representational capacity of key regions. (C) The classification module. The fully connected layer and the softmax classifier are used to obtain the predicted level of seed setting and anther emergence. (D) Details of feature extraction module. The implementation details of the modules of Stem, Reduction-A and Reduction-B. These modules achieve multi-scale feature propagation and dimension compression through combining the convolution and max pooling operations.

This study proposes Maize-IRNet, a grading evaluation model for haploid anther emergence and ear seed setting based on the Inception-ResNet architecture and a global attention mechanism. As shown in Fig. 2, Maize-IRNet mainly includes three modules: Inception-ResNet, Reduction, and GAM. Haploid fertility images contain fine-grained features of different scales and levels, such as varying anther distributions and densities in tassels. To this end, the Inception-ResNet module in Maize-IRNet effectively captures multi-scale fine-grained features using a multi-scale network and residual mechanisms. The Reduction module enhances the representation of complex phenotypes by optimizing cross-scale information transfer. In addition, the global attention mechanism can effectively enhance the model's ability to focus on key regions by modeling the dependencies between channels and spatial domains. By leveraging this mechanism, Maize-IRNet pays more attention to the anther regions in tassels, and provides a more comprehensive representation of the overall grain distribution in ears.

### 2.4 Backbone of Maize-IRNet

The backbone network of Maize-IRNet is implemented based on the Inception-ResNet architecture [37], which captures trait information of maize fertility restoration images by extracting multi-scale features. Inception-ResNet combines the multi-path parallel convolution structure of the Inception network [[34], [35], [36], [37]] with the residual connections of ResNet [38], enabling the extraction of richer and more hierarchical key region features of different scales. The backbone network consists of multiple functional modules, including the Stem module, Inception-ResNet-A module, Reduction-A module, Inception-ResNet-B module, and Reduction-B module.

Stem module: The Stem module efficiently extracts initial image features through the convolution operations (*Conv*) while avoiding excessive dimension expansion of feature map at an early stage. Specifically, this module firstly extracts preliminary image features by stacking three sets of 3 ​× ​3 convolution layers, and the convolution operation is defined in Eq. (1). After each convolution layer, Batch Normalization (BN) and the ReLU activation function are used to enhance feature representation. Then the 3 ​× ​3 max pooling layer (*Maxpool*) is used to compress spatial dimensions. Furthermore, an *ERF* submodule is employed to expand the receptive field of feature extraction. Firstly, 1 ​× ​1 convolution is used to adjust the number of channels to optimize feature representation. Then, two sets of 3 ​× ​3 convolution layers are used to deepen feature extraction. The final output is a feature map *X*_1_ with the size of 35 ​× ​35 ​× ​256. The feature map contains rich multi-scale local features and global contextual information, laying the foundation for deep feature fusion in subsequent modules. The implementation of *ERF* module is shown in Eq. (2), where *X* represents the input image features, *i* denotes the output position index, *k* is the convolution kernel index, and the convolution kernel *W* is an *M×N* matrix. *Conv1* and *Conv3* refer to the convolution operations with different kernel sizes. The implementation of the Stem module is shown in Eq. (3).

(1) $$Conv\left(X\right)=ReLU\left\{\sum_{m=0}^{M-1}\sum_{n=0}^{N-1}W_{m,n}^{k}X_{i*step+m,n}\right\}$$

(2) $$\mathrm{ERF}\left(X\right)=Conv_{3}\left(Conv_{3}\left(Conv_{1}\left(X\right)\right)\right)$$

(3) $$X_{1}=\mathrm{ERF}(Maxpool\left(Conv_{3}\left(Conv_{3}\left(Conv_{3}\left(X\right)\right)\right)\right)$$

Inception-ResNet-A/B Modules: The modules of Inception-ResNet-A and Inception-ResNet-B are the core components of Maize-IRNet. The two modules extract image features by integrating Inception structures and residual connections, which have the characteristics of optimizing gradient flow and accelerating network training. These modules can be flexibly stacked to enhance feature extraction by increasing network depth. Each module employs multiple parallel convolutional branches to capture features at different scales. Inception-ResNet-A includes three parallel branches: Branch 1 uses a 1 ​× ​1 convolution to perform channel-wise feature transformation and retain fine-grained details of the original feature map. Branch 2 uses a combination of 1 ​× ​1 and 3 ​× ​3 convolutions to capture medium-scale features. Branch 3 extracts larger-scale contextual information based on the *ERF* module. Finally, the outputs of all the branches are concatenated (*concat*) and fused using a 1 ​× ​1 convolution to integrate channel information. The extracted multi-scale features are then added to the input features through a residual connection, followed by a ReLU activation function to obtain the final feature representation of the module (Eq. (4)).

(4) $$X_{2}=ReLU\left(X_{1}+Conv_{1}\left(concat\left(\begin{array}{l} Conv_{1}\left(X_{1}\right)\\ Conv_{3}\left(Conv_{1}\left(X_{1}\right)\right)\\ \mathrm{ERF}\left(X_{1}\right) \end{array}\right)\right)\right)$$

The structure of Inception-ResNet-B is similar to that of Inception-ResNet-A. The key difference is that Inception-ResNet-B introduces more asymmetric convolutional kernels, enabling better capture long-distance dependencies. This module includes two parallel branches: Branch 1 uses a 1 ​× ​1 convolution for channel-wise feature transformation, and Branch 2 utilizes a combination of 1 ​× ​1, 1 ​× ​7 and 7 ​× ​1 asymmetric convolutions to decompose large kernels. These operations can effectively reduce computational complexity and capture long-distance spatial context information in the images. Additionally, both Inception-ResNet-A and Inception-ResNet-B maintain consistent spatial dimensions between the input and output feature maps, facilitating stacking of multiple modules.

Reduction-A/B module: The Reduction module is located after two Inception ResNet modules, and it is used to integrate multi-scale features. By combining a multi-branch architecture with downsampling, Reduction-A module can reduce the spatial resolution of the feature maps while retaining the integrity of semantic information. This module processes the input features through three distinct branches (3 ​× ​3 convolution, *ERF* module, and max pooling operation) and then concatenates the outputs of these branches. Reduction-A outputs a feature map *X*_3_ with the size of 17 ​× ​17 ​× ​896, as shown in Eq. (5).

(5) $$X_{3}=concat\left(\begin{array}{l} Conv_{3}\left(X_{2}\right)\\ \mathrm{ERF}\left(X_{2}\right)\\ Maxpool\left(X_{2}\right) \end{array}\right)$$

The Reduction-B module includes four branches. Branches 1 and 2 extract local features using 1 ​× ​1 and 3 ​× ​3 convolutions to achieve efficient downsampling. Branch 3 uses *ERF* module structure to model long-range dependencies and capture contextual information. Branch 4 employs max pooling to preserve high-level semantic features and ensure that the crucial structural information is retained. This architecture not only ensures the richness of feature extraction but also optimizes computational efficiency through the balance of channel expansion and spatial downsampling. This module is particularly suited for capturing complex phenotype features in images, thereby improving prediction accuracy.

### 2.5 Global attention mechanism

By introducing channel attention and spatial attention, the Global Attention Mechanism (GAM) [39] enhances the model's selective focus on key features, as shown in Fig. S1. By learning and adjusting feature weights, this mechanism optimizes features in the channel and spatial domains separately. It captures global interrelationships in the channel domain and emphasizes critical feature regions in the spatial domain, thereby enabling finer-grained feature extraction. This dual attention mechanism enhances the expression of key features, generate feature representations that are easier to distinguish different classes, and ultimately improve the accuracy of classification tasks. GAM firstly employs the channel attention mechanism to assign different weights to features in each channel, thus to enhance the attention to important channels. Specifically, the channel attention mechanism firstly rearranges the C ​× ​H ​× ​W feature map into H ​× ​W ​× ​C through *permute* operation, and then uses *MLP*_1_ to the permuted feature map for feature learning. This process not only compresses the number of channels and reduces computational complexity, but also captures the feature representation of each channel effectively. After the ReLU activation, the module uses an inverse transformation to restore the feature map to its original size of C ​× ​H ​× ​W. Finally, the module uses *Sigmoid* function to compress the output values into the range of (0,1), thereby generating the channel attention weight matrix. The implementation of channel attention mechanism is shown in Eq. (6), where *c* denotes the number of channels and *rate* denotes the compression ratio of channel numbers.

(6) $$X_{channel}=Sigmoid\left(Repermute\left(MLP_{2}\left(ReLU\left(MLP_{1}\left(Permute\left(X\right),\frac{c}{rate}\right)\right),c\right)\right)\right)$$

The spatial attention mechanism enhances the focus on critical spatial regions by assigning weights to different positions in the feature map. This module firstly employs 7 ​× ​7 convolution and *ReLU* activation to extract spatial information and adjust the channel numbers. Then, it uses the second convolution layer to process the spatial features and restores the channel number to its original size. Finally, the *Sigmoid* function is used to generate spatial attention matrix, and reweights each spatial location. The implementation of the spatial attention mechanism is shown in Eq. (7).

(7) $$X_{spatial}=Sigmoid\left(Conv_{7}\left(ReLU\left(Conv_{7}\left(X\right)\right)\right)\right)$$

Finally, GAM employs element-wise multiplication to progressively adjust the importance of each channel and spatial location based on the spatial and channel attention maps, thereby enhancing the capability of capturing the discriminative features. The implementation of GAM is shown in Eq. (8).

(8) $$X_{out}=X⊗X_{channel}⊗X_{spatial}$$

### 2.6 Experimental details

To comprehensively train and test the performance of the proposed Maize-IRNet model, we designed and conducted a series of experiments. The experimental environment was configured as follows: the hardware platform consists of an AMD EPYC 9754 ​128-Core Processor@2.25 GHz with 1 ​TB DDR5 memory and an NVIDIA GeForce RTX 4090 GPU. The software environment was deployed on Ubuntu Linux 22.04.3 LTS operating system, with key dependencies including Python 3.9.18 interpreter, PyTorch 2.0.0 deep learning framework, and configured with CUDA 11.8 parallel computing platform and cuDNN 8.7 deep neural network acceleration library to support GPU computing.

The input data of all the models were subjected to unified preprocessing (such as denoising, standardization, *etc*.) and data augmentation operations. The Cross-Entropy loss function was used to quantify the discrepancy between the predicted probability distribution and ground-truth labels. The implementation of Cross-Entropy loss is shown in Eq. (9), where *n* denotes the number of samples and *k* represents the number of classes. *y*_*ij*_ denotes the true label for the *i*-th sample belonging to the *j*-th class, and *p*_*ij*_ represents the predicted probability that the *i*-th sample belongs to the *j*-th class.

(9) $$Loss_{BCE}\left(n,k\right)=-\frac{1}{n}\sum_{i=1}^{n}\sum_{j=1}^{k}\left[y_{ij}\;\log\left(p_{ij}\right)\right]$$

The Adam optimizer can adaptively adjust the learning rate of each parameter while maintaining stability during the optimization process [40]. It can effectively mitigate the problems of gradient explosion and gradient disappearance in most cases. We employ the Adam optimizer to update the parameters, and it includes the following steps.

1 The Adam optimizer firstly computes the first-order moment (Eq. (10)) and second-order moment (Eq. (11)) of gradients. *β*_*1*_ and *β*_*2*_ denote the decay rates of the first and second moments, respectively, and *g*_*t*_ represents the gradient calculated at the *t*-th iteration.

(10) $$m_{t}=\beta_{1}m_{t-1}+\left(1-\beta_{1}\right)g_{t}$$

(11) $$v_{t}=\beta_{2}v_{t-1}+\left(1-\beta_{2}\right)g_{t}^{2}$$

2. Adam employs the Exponentially Weighted Moving Average (EWMA) method to estimate the first-order and second-order moment. If the initial values of the two moments are set to zero (*m*_*0*_ ​= ​0, *v*_*0*_ ​= ​0), the expected values of *m*_*t*_ and *v*_*t*_ can significantly deviate from the true gradient statistics in the early iterations. To this end, Adam optimizer uses the bias correction factors 1/(1-*β*^*t*^) to transform the raw moment estimates into unbiased estimators. The bias correction of the first-order and second-order moment is shown in Eq. (12) and Eq. (13).

(12) $${\hat{m}}_{t}=\frac{m_{t}}{1-\beta_{1}^{t}}$$

(13) $${\hat{v}}_{t}=\frac{v_{t}}{1-\beta_{2}^{t}}$$

3. The optimizer updates the parameters using the corrected values, as shown in Eq. (14), and *α* represents the global learning rate and *ϵ* is a numerical stability constant to avoid the problem of zero division calculation when the gradient second-order moment estimation approaches zero. Moreover, the parameter *ϵ* helps to mitigate the drastic changes in parameter update amplitude caused by inadequate estimation of the gradient variance.

(14) $$\theta_{t+1}=\theta_{t}-\frac{\alpha {\hat{m}}_{t}}{\sqrt{{\hat{v}}_{t}}+\varepsilon }$$

During model training, we use the steady decrease in the loss function as the criterion. We set the Batchsize to 16 and set the number of Epochs to 300. The global learning rate is initially set to *lr*_*max*_ ​= ​10^−4^ and it is gradually adjusted to *lr*_*min*_ ​= ​10^−6^ through Cosine annealing strategy [41]. The Cosine annealing strategy helps the model to gradually reduce the learning rate in the later stages of training, thereby mitigating overfitting. Additionally, the Cosine annealing strategy can avoid the learning rate early dropping to zero, which could lead to the optimization process to stagnate. Its update rule is shown in Eq. (15), where *t* denotes the iteration number in the current cycle and *T*_max_ represents the length of a complete cosine annealing cycle.

(15) $$lr\left(t\right)=lr_{\min}+\frac{1}{2}\left(lr_{\max}-lr_{\min}\right)\left(1+\cos\left(\frac{t}{T_{\max}}\pi \right)\right)$$

### 2.7 Evaluation metrics

We used the following metrics to evaluate the model's performance in the classification task: Accuracy, Precision, Recall, F1-Score, and the confusion matrix. The confusion matrix could clearly illustrate the concrete information of the actual classes and the predicted classes, thereby reflecting the classifier performance. It includes four elements: True Positives (TP), True Negatives (TN), False Positives (FP), and False Negatives (FN).

Accuracy can measure the proportion of the correctly classified samples relative to the total number of samples, as shown in Eq. (16).

(16) $$Accuracy=\frac{TP+TN}{TP+TN+FP+FN}$$

Recall rate is used to calculated the proportion of the actual positive samples that are correctly identified by the classifier, as shown in Eq. (17).

(17) $$Precision=\frac{TP}{TP+FP}$$

Precision denotes the proportion of samples predicted as positive that are actually positive, as shown in Eq. (18).

(18) $$Recall=\frac{TP}{TP+FN}$$

The F1-Score represents a balanced measure between Precision and Recall rate. It is used to comprehensively evaluate the performance of the classifier and it is suitable for situations with imbalanced categories. The calculation process of F1-Score is shown in Eq. (19).

(19) $$F1-Score=2*\frac{Precision*Recall}{Prescision+Recall}$$

### 2.8 Comparison methods

To evaluate the effectiveness of Maize-IRNet, we compared it with the classical deep learning models of Visual Geometry Group (VGG) [42], Residual Network (ResNet) [38], Vision Transformer (ViT) [43], MLP-Mixer [44], and gMLP [45]. These models have demonstrated better performance in computer vision tasks and have already achieved promising results in crop image processing applications. Among them, ResNet excels in various tasks due to its deep architecture and the advantages of residual connections learning. The residual connections can effectively mitigate the vanishing gradient problem and enable learning more complex feature representations. For instance, some studies have combined Grad-CAM with ResNet50 to accurately capture key phenotypic features of leaves [46]. VGG is widely used in image processing due to its simple network architecture and robust feature extraction capability, with its stacked 3 ​× ​3 convolution layers effectively capturing local image features. Based on the transformer architecture, ViT partitions images into multiple patches and uses self-attention mechanisms to capture global contextual information, thereby overcoming the limitations of local receptive fields about the traditional convolutional neural networks. Researchers have designed lightweight improved ViT models to achieve high-accuracy recognition of maize diseases [47]. As novel deep learning models based on multilayer perceptrons (MLP), MLP-Mixer and gMLP extract features though global feature interaction and channel mixing mechanisms, avoiding the traditional convolutional operations and demonstrating significant potential in agricultural image analysis.

## 3 Results

### 3.1 Grading classification results of tassel anther emergence and ear seed setting

We randomly divided the dataset into training set (90 ​%) and testing set (10 ​%) for experimental comparison. Table 1 shows the performance metrics for evaluating the classification of haploid ear seed setting rate and tassel anther emergence rate using different methods. It can be seen that Maize-IRNet achieved better accuracy (84.2 ​%), recall rate (83.7 ​%), precision (83.8 ​%), and F1-score (83.7 ​%). The accuracy of VGG11_bn and ResNet50 is 81.5 ​%, and ResNet101 achieved an accuracy of 81.0 ​%. The other three metrics of these models are also lower than those of Maize-IRNet. The accuracy of ViT-Base-16 and MLP-Mixer is 76.8 ​%, and gMLP achieved an accuracy of 78.4 ​%. For the haploid tassel anther emergence grading, Maize-IRNet achieved an accuracy of 84 ​%, recall rate of 75.3 ​%, precision of 74.3 ​%, and F1-score of 74.8 ​%. When compared to widely used CNN architectures, Maize-IRNet demonstrated superior accuracy over VGG11_bn (82.9 ​%) and ResNet-based models (ResNet50: 80.9 ​%; ResNet101: 79.8 ​%). In contrast, with the newer architectures, Maize-IRNet exhibited an accuracy improvement ranging from 10.3 ​% to 23 ​% compared to ViT-Base-16 (61 ​%), gMLP (72.7 ​%), and MLP-Mixer (68.2 ​%). Overall, despite each of the other six methods displaying certain strengths in individual performance metrics, Maize-IRNet's indicators are significantly higher, demonstrating the best overall prediction performance. Overall, although the other six methods have their own strengths in terms of accuracy, recall rate, precision, and F1-score, these indicators of Maize-IRNet are significantly higher than the other six methods.

**Table 1:** Experimental results of the classification of haploid ear seed setting rate and tassel anther emergence rate.

Dataset	Methods	Accuracy	Recall rate	Precision	F1-Score
Ear seed setting rate	VGG11_bn	81.5	80.6	80.8	80.7
	ResNet50	81.5	80.4	81.4	80.9
	ResNet101	81.0	79.6	80.3	79.9
	ViT-Base-16	76.8	76.4	76.9	76.6
	gMLP	78.4	76.9	76.9	76.9
	MLP-Mixer	76.8	75.1	75.8	75.4
	**Maize-IRNet**	**84.2**	**83.7**	**83.8**	**83.7**
Tassel anther emergence rate	VGG11_bn	82.9	72.8	73.0	72.9
	ResNet50	80.9	69.8	71.6	70.7
	ResNet101	79.8	70.2	71.5	70.8
	ViT-Base-16	61.0	41.9	38.5	40.1
	gMLP	72.7	63.3	60.3	61.8
	MLP-Mixer	68.2	55.2	55.2	55.2
	**Maize-IRNet**	**84.0**	**75.3**	**74.3**	**74.8**

**Fig. 3:**
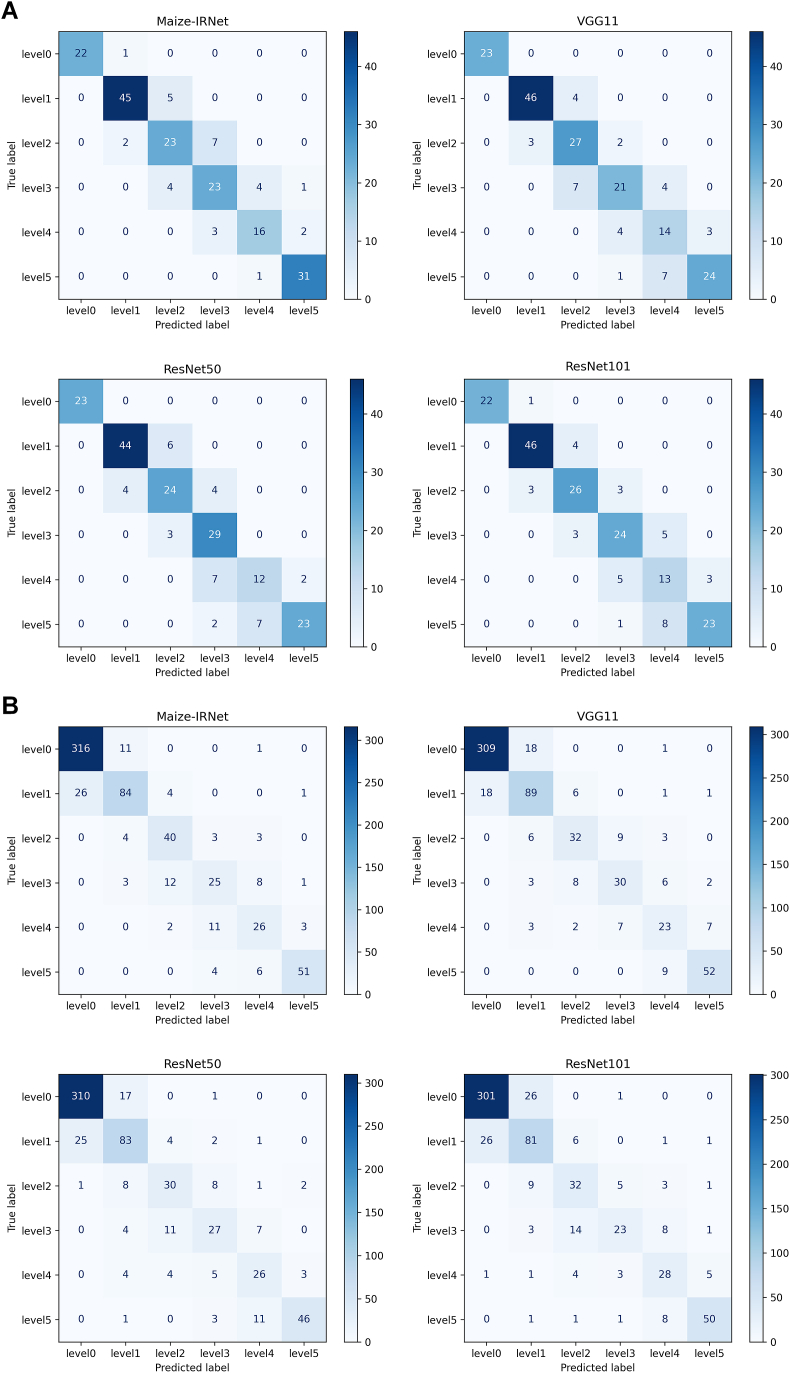
Confusion matrices for grading predictions using different models. (A) The grading confusion matrix for haploid ear seed setting rate. (B) The grading confusion matrix for tassel anther emergence rate.

The above results indicate that Maize-IRNet, VGG11_bn, ResNet50, and ResNet101 have better performance on the test set of the ear seed setting rate. Fig. 3A elaborates the confusion matrices for prediction results of these four models. The result shows that Maize-IRNet has a significant advantage in classifying the samples with level 4 and level 5, resulting in its classification accuracy being apparently superior to that of the other models. Fig. 3B shows the confusion matrices for the better-performing classification models on the tassel anther emergence grading. It can be seen that Maize-IRNet achieves better classification performance across multiple levels, particularly exhibiting a lower misclassification rate in the level 2. These results indicate that Maize-IRNet is able to capture the key features of ear images more effectively, thereby demonstrating superior performance in the complex classification tasks. The confusion matrices of the other three comparative methods are shown in Fig. S2 and Fig. S3.

### 3.2 Experimental results of data augmentation

To evaluate the generalization capability and robustness of the model, we conducted data augmentation experiments on the original dataset. Each image was transformed by adding Gaussian noise, adjusting brightness, and introducing blur effects, resulting in three variants for each sample. We retrained the model using the augmented dataset, and the results are shown in Table 2. It can be seen that Maize-IRNet still maintained the highest accuracy although the classification accuracy of some models declined after data augmentation. These results indicate that Maize-IRNet possesses better generalization capabilities and interference resistance, enabling it to maintain stable classification performance in complex noisy conditions.

**Table 2:** Model performance comparison of data augmentation.

Dataset	Methods	Accuracy	Recall rate	Precision	F1-Score
Ear seed setting rate	VGG11_bn	81.3	79.9	79.9	79.9
	ResNet50	80.3	78.7	78.3	78.5
	ResNet101	81.3	79.1	79.4	79.2
	ViT-Base-16	80.8	79.5	79.8	79.6
	gMLP	81.3	78.9	79.4	79.1
	MLP-Mixer	78.7	77.4	78.5	77.9
	**Maize-IRNet**	**81.9**	**80.5**	**81.1**	**80.8**
Tassel anther emergence rate	VGG11_bn	79.7	68.6	69.9	69.2
	ResNet50	75.1	65.1	64	64.5
	ResNet101	72.4	60.0	62.1	61.0
	ViT-Base-16	67.1	53.2	54.7	53.9
	gMLP	64.4	52.2	53.7	52.9
	MLP-Mixer	67.4	53.4	55.0	54.2
	**Maize-IRNet**	**80.7**	**71.3**	**72.0**	**71.6**

### 3.3 Ablation experiments of Maize-IRNet

To evaluate the impact of distinct foundational components within Maize-IRNet network on grading prediction results, we conducted a series of ablation experiments. In one set of experiments, based on the original backbone of Maize-IRNet, we compared the grading performance after removing different Inception-ResNet modules and substituting the Reduction module (Table 3). Without removing any modules, Maize-IRNet backbone achieves an accuracy of 82.1 ​%, recall rate of 81.8 ​%, precision of 81.4 ​%, and F1-score of 81.6 ​%. When the Inception-ResNet-A module is removed, the model accuracy drops to 79.5 ​% and F1-score decreases to 78.9 ​%. Removal of the Inception-ResNet-B module leads to an even more pronounced decrease in performance, with accuracy of 78.4 ​%. It indicates that the Inception-ResNet modules play a critical role in capturing complex feature patterns, and their absence significantly impairs the model's ability to extract high-level features, thereby affecting classification performance. In addition, replacing both Reduction modules with stride-2 3 ​× ​3 convolutions will result in reduced accuracy (81.1 ​%, 80.5 ​%), indicating that Reduction modules contribute to the optimized feature representation. These ablation experiment results demonstrate that each component of the backbone independently contributes to the overall performance, and the removal of any module leads to a decline in prediction accuracy. The ablation experimental results of tassel anther emergence rate are shown in Table S2.

**Table 3:** Ablation and attention mechanisms integrating results of Maize-IRNet on haploid ear seed setting rate.

Experiments	Method	Metrices			
Ablation of backbone	Remove module	Accuracy	Recall rate	Precision	F1-Score
	Inception-resnet-A	79.5	79.8	78.0	78.9
	Reduction-A	81.1	80.0	80.0	80.0
	Inception-resnet-B	78.4	77.2	78.4	77.8
	Reduction-B	80.5	80.6	80.5	80.5
	**Backbone**	**82.1**	**81.8**	**81.4**	**81.6**
Comparison of integrated different attention modules	Attention module	Accuracy	Recall rate	Precision	F1-Score
	Backbone	82.1	81.8	81.4	81.6
	CBAM	80.0	78.2	78.5	78.3
	SE	80.0	77.9	78	77.9
	EMA	81.5	80.1	81.4	80.7
	**GAM**	**84.2**	**83.7**	**83.8**	**83.7**

In order to systematically evaluate the impact of the global attention mechanism on model performance, we integrated four mainstream attention modules into the backbone network for comparison experiments: Convolutional Block Attention Module (CBAM) [48], Squeeze-and-Excitation (SE) [49], Efficient Multi-Scale Attention (EMA) [50], and the proposed GAM. We compared the effects of these four attention modules on the prediction performance, and the results are shown in Table 3. It can be seen that the GAM module is important for the prediction performance, as it improves the model accuracy from the baseline of 82.1 ​%–84.2 ​%, and the F1-score from 81.6 ​% to 83.7 ​%. These results validate the superiority of the GAM module in optimizing feature representations. In contrast, the introduction of other attention mechanisms did not improve prediction accuracy. Specifically, integrating the CBAM module reduced the accuracy to 80.0 ​%, while the incorporation of SE and EMA modules did not significantly improve model performance. These experimental results illustrate the effectiveness and necessity of the GAM module in Maize-IRNet, particularly the crucial role of attention mechanisms in boosting model performance. The ablation experimental results of integrating four attention mechanisms for predicting anther emergence rate are shown in Table S3.

### 3.4 Comparison analysis of different initialization methods

To investigate the impact of model initialization on the classification performance of Maize-IRNet, we conducted a series of experiments with different initialization methods. We compared four mainstream parameter initialization strategies while ensuring that the network structure remained unchanged: random initialization (Normal) based on normal distribution, Xavier initialization [51], Orthogonal initialization [52], and He initialization [53]. Among them, Xavier initialization mitigates the gradient vanishing problem by setting the variance of the weights, making it suitable for the activation functions of Sigmoid and Tanh. Orthogonal initialization reduces redundant parameters and improve training stability through maintaining the geometric structure of the feature space. He initialization is mainly optimizing the ReLU activation by adjusting the variance to adapt the characteristics of ReLU, and it tends to perform better in deep networks. The experimental results are shown in Table S4.

The results indicate that He initialization achieved the best prediction performance, with an accuracy of 84.2 ​% and F1-score of 83.7 ​%. This result confirms that He initialization is particularly suited for deep network architectures including the ReLU activation functions. As a baseline for comparison, Random initialization obtains an accuracy of 83.2 ​% and F1-score of 82.8 ​%, providing a reliable reference for evaluating other initialization methods. Although Xavier initialization also results in performance improvements, the accuracy improvement is relatively small. Xavier initialization has an accuracy of 83.6 ​% and F1-score of 83.0 ​%. Orthogonal initialization has the lowest prediction performance, with an accuracy of 83.1 ​% and F1-score of 82.5 ​%. These experimental results highlight the impact of different initialization methods on the performance of Maize-IRNet and offer important insights for the selection of initial parameters.

### 3.5 Experimental results on balanced sample data

The sample sizes of tassel anther emergence and ear seed setting in different levels are shown in Table S5. It can be seen that the number of tassel images in level 0 and level 1 is significantly larger than that of the other categories. For ear images, the sample sizes from level 0 to level 5 are relatively balanced, with only level 1 containing slightly more samples than the others. To investigate the effect of class imbalance on the prediction performance of Maize-IRNet, we conducted fertility restoration grading experiments using a balanced dataset. The balanced dataset was obtained by downsampling the categories with excessively large sample sizes. Specifically, 500 samples were randomly selected from each of level 0 and level 1 of the tassel anther emergence datasets, and 300 samples were randomly selected from level 1 of the ear seed setting dataset. The grading accuracy of haploid ear seed setting rate and tassel anther emergence rate achieved by different methods on the balanced dataset is shown in Table 4. Maize-IRNet outperformed the classical models of VGG11_bn, ResNet50, and ResNet101 in most indicators, including accuracy, recall rate, precision, and F1-score. In addition, the four evaluation indicators of Maize-IRNet show no significant difference before and after data balancing. It indicates that Maize-IRNet is highly robust, and its prediction performance is not affected by unbalanced distribution of categories.

**Table 4:** The ear seed setting and tassel anther emergence grading evaluation results of different methods under balanced sample size and comparison results on the independent test set.

Experiments	Dataset	Methods	Accuracy	Recall rate	Precision	F1-Score
Comparison under balanced sample size	Ear seed setting rate	VGG11_bn	79.5	78.6	78.3	78.4
		ResNet50	79.5	79.1	78.8	78.9
		ResNet101	78.9	78.0	77.4	77.7
		**Maize-IRNet**	**81.5**	**79.8**	**80.1**	**79.9**
	Tassel anther emergence rate	VGG11_bn	80.9	72.4	72.4	72.4
		ResNet50	75.5	67.0	66.9	66.9
		ResNet101	74.8	65.9	64.7	**65.3**
		**Maize-IRNet**	**81.2**	**72.1**	**73.2**	**72.6**
Comparison on the independent test set	Ear seed setting rate	VGG11_bn	80.3	77.5	78.9	78.2
		ResNet50	78.2	76.1	77.1	76.6
		ResNet101	79.8	77.0	77.4	77.2
		**Maize-IRNet**	**82.4**	**81.0**	**81.7**	**81.3**
	Tassel anther emergence rate	VGG11_bn	82.2	69.3	71.4	70.3
		ResNet50	79.4	67.9	68.6	68.2
		ResNet101	77.5	64.8	66.9	65.8
		**Maize-IRNet**	**82.6**	**72.6**	**72.6**	**72.6**

To explore the effect of different data partitioning strategies on prediction performance, we conducted experiments of ten-fold cross-validation and independent test set evaluation. To reduce the instability caused by random data splitting, we firstly evaluated the performance of Maize-IRNet using ten-fold cross-validation (Table S6). The prediction accuracy of cross-validation showed no significant difference compared with that of random splitting, indicating that Maize-IRNet has strong robustness. In addition, we divided the dataset into training set (80 ​%), validation set (10 ​%), and independent test set (10 ​%). The results are shown in Table 4. For ear seed setting rate, the accuracy, recall rate, precision, and F1-score of Maize-IRNet on the independent test set were 82.4 ​%, 81.0 ​%, 81.7 ​%, and 81.3 ​%. In comparison, the accuracies of ResNet50, ResNet101, and VGG11_bn were 78.2 ​%, 79.8 ​%, 80.3 ​%, respectively. The recall rate, precision, and F1-score of Maize-IRNet also outperformed those of the comparison models. For tassel anther emergence rate, Maize-IRNet achieved an accuracy of 82.6 ​%, with recall, precision, and F1-score all at 72.6 ​%. The best baseline, VGG11_bn, reached an accuracy of 82.2 ​%, still lower than Maize-IRNet. These results of multiple data partitioning strategies demonstrate that Maize-IRNet achieves the highest prediction accuracy and exhibits strong robustness.

### 3.6 Model performance and computational efficiency of different resolution images

To explore the effect of different image resolution on the prediction performance of Maize-IRNet, we compared the prediction accuracy of the experiments of 2048 ​× ​2048 high-resolution images and 299 ​× ​299 resolution images. The results are shown in Table S7. Specifically, the training time is defined as the average running time of each epoch. The experimental results indicate that Maize-IRNet achieved nearly the same prediction accuracy of the above two resolutions. In terms of resource consumption, the runtime and GPU memory usage of 2048-pixel high-resolution images were at least four times and ten times higher than those of 299-pixel resolution images, respectively. This suggests that downscaling 2048-pixel high-resolution images to 299-pixel resolution does not lead to information loss. Moreover, Maize-IRNet required only 2.9 ​GB of GPU memory to process 299-pixel resolution images, leaving sufficient space for larger hyperparameter tuning and model optimization. Therefore, Maize-IRNet maintains high prediction accuracy and achieves a better balance between computational efficiency and performance when to process the images into 299-pixel resolution.

### 3.7 Interpretability analysis of Maize-IRNet

**Fig. 4:**
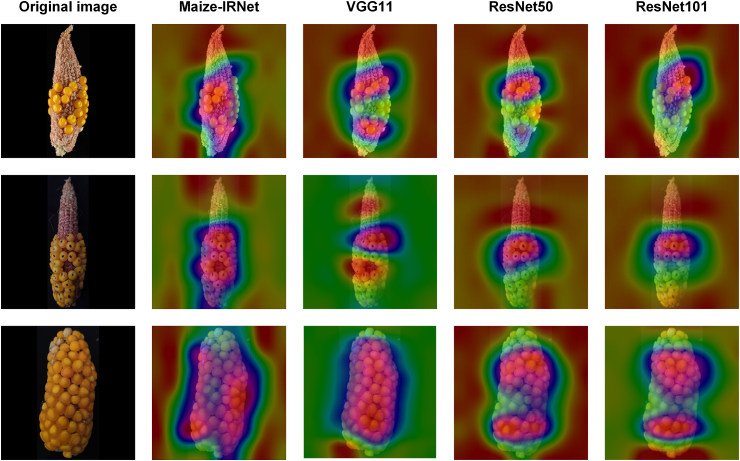
Feature attention heatmap of VGG11_bn, ResNet50, ResNet101 and Maize-IRNet.

In order to deeply analyze the feature learning ability and attention to key regions of Maize-IRNet, we used Gradient-weighted Class Activation Mapping (Grad-CAM) [54] method to visualize decision-making process of the model. By extracting gradient information from the convolutional layers, we generated class activation heatmaps to visually demonstrate the key regions that different models focus on during prediction. We selected three representative maize images with different seed setting rates (level 3-level 5) for analysis. Fig. 4 shows the visualization results of Maize-IRNet, VGG11_bn, ResNet50, and ResNet101. The results indicate that Maize-IRNet can accurately capture critical features, with its heatmap covering the main regions of the seeds in a uniform manner and particularly emphasizing areas with concentrated seeds. In contrast, VGG11_bn, ResNet50, and ResNet101 exhibit a more focus on local regions, potentially omitting some important features of the ear. The feature attention heatmap of VGG11_bn, ResNet50, ResNet101 and Maize-IRNet in tassel anther emergence rate grading is shown in Fig. S4.

**Fig. 5:**
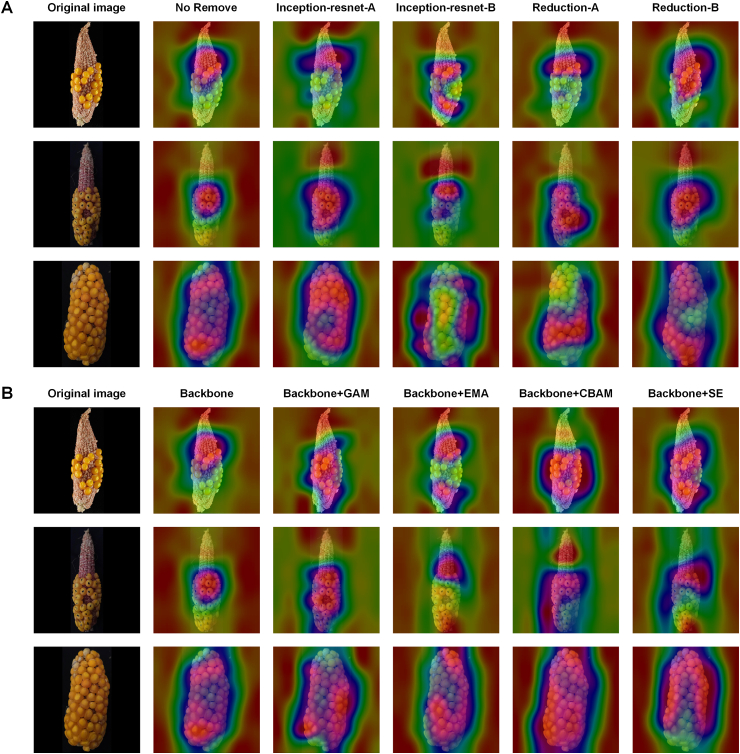
Feature attention heatmaps for (A) backbone network and (B) attention modules in the ablation experiments.

To further investigate the effectiveness of different modules in Maize-IRNet for capturing key feature regions, we analyzed the visualization results of the backbone network in the ablation experiments, as shown in Fig. 5A. It can be seen that the network extracts feature over a broad area when no module is removed. Maize-IRNet demonstrates comprehensive attention to the haploid ear, with a more uniformly distributed heatmap. The model can more effectively capture the critical seed regions, thereby exhibiting excellent capability of capturing global features. After removing the two Inception-ResNet modules, there are some deviations in the area and position of the attention region that Maize-IRNet focuses on. Furthermore, when the Reduction module is replaced, there is a significant loss of focus on images with high seed setting rate, indicating that the Reduction module plays an important role in preserving key information and optimizing feature representations. These results further demonstrate that the modules in Maize-IRNet work synergistically to enhance the model's feature extraction ability and classification performance. Any removal or replacement of a module results in a decline in predictive performance, further validating the rationality of the network architecture and module configuration.

In addition, we evaluate the impact of different attention mechanisms on the feature extraction of Maize-IRNet using the visualization analysis experiments of the attention modules. The heatmap of key region features captured by different attention modules is shown Fig. 5B. It can be seen that the GAM module demonstrates superior performance in capturing key feature regions, with its focus precisely concentrated on seed dense areas. The GAM module exhibits higher feature activation intensity, indicating its effectiveness in capturing crucial local features. In contrast, other attention modules (e.g., EMA and SE) result in a more dispersed feature concentration in the seed regions and weaken the overall activation intensity, suggesting limitations in extracting local features. Although the CBAM can effectively localize target regions, it produces distracting activation responses when identifying non-target regions (e.g., areas without seeds), leading to inferior performance compared with GAM.

### 3.8 Comparison analysis of computational efficiency

We also compared the parameter quantity and running time of the seven models. All the numbers of parameters are calculated based on float32 (single precision, 4 bytes per parameter), and the experimental results are shown in Table S8. Owing to the incorporation of the Global Attention Mechanism (GAM), Maize-IRNet has a parameter size of 137.15 ​MB. Although the parameter size of Maize-IRNet is higher than the lightweight models of gMLP (11.33 ​MB) and MLP-Mixer (80 ​MB), it is still significantly lower than that of VGG11_bn (491.32 ​MB) and ViT-Base-16 (326.73 ​MB). The high parameter quantity of VGG11_bn is mainly related to the complex structure of its fully connected layers. ViT-Base-16 has a greater number of parameters due to the stacking of encoder layers and high-dimensional linear projections. In contrast, Maize-IRNet combines its base architecture with GAM to maintain a relatively reasonable parameter scale while still ensuring strong performance. For the running efficiency, we compared the time taken by each model to complete a single forward propagation. The results show that Maize-IRNet achieves an inference time of 0.00663 ​s, which is faster than the multi-parameter model of ViT-Base-16 (0.02616 ​s) but slower than the lightweight model of gMLP (0.00158 ​s). These findings suggest that Maize-IRNet not only offers superior inference efficiency compared to the high-parameter models but also strikes a balance between computational efficiency and the model's representational capability. Notably, the compression ratio of the channel attention in GAM module is a crucial factor affecting both the number of parameters and the inference time. In this experiment, the compression ratio *rate* is set to 16 in Maize-IRNet. It can ensure the effectiveness of the attention mechanism, limit the increase in additional parameters, thereby keeping the inference time within a reasonable range.

### 3.9 Develop a mobile application of haploid-fertility

**Fig. 6:**
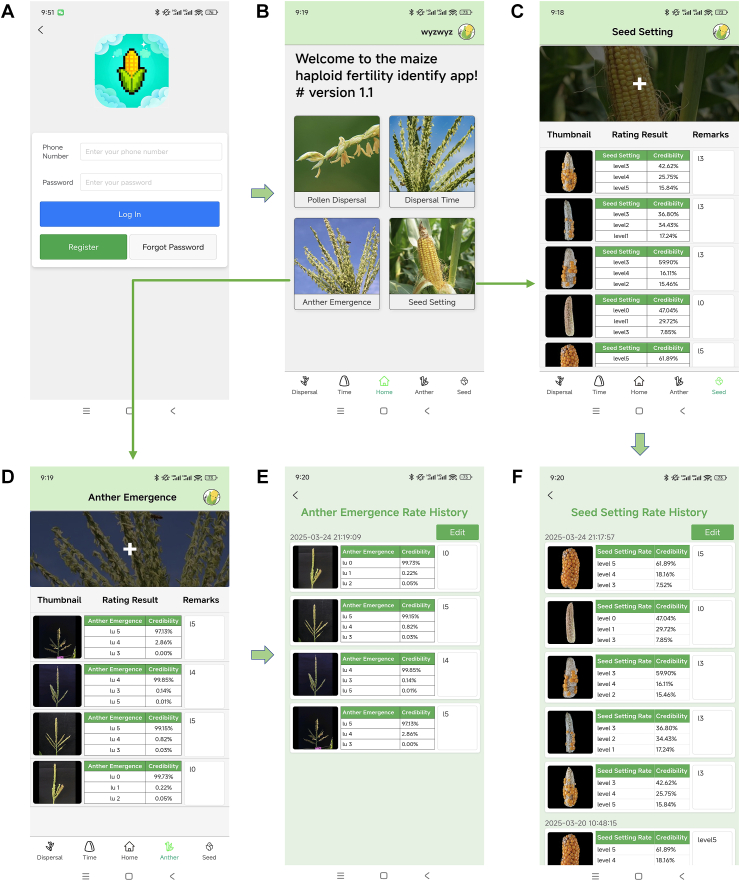
Main interfaces of Haploid-Fertility. (A) The login page. Users can login and use the model to obtain prediction results. (B) The startup page. (C) The result page of seed setting rate prediction. Displays prediction results for the submitted images in a tabular format. (D) The usage page of anther emergence rate evaluation. Grading prediction results of anther emergence rate. (E) Anther emergence prediction history page. Users can review, edit, and export historical prediction results of multiple models. (F) Seed setting prediction history page.

To facilitate the practical usage of Maize-IRNet by breeders and researchers, we developed a user-friendly mobile application (Haploid-Fertility) for evaluating fertility restoration levels of maize haploids. The backend architecture is the widely used Spring Boot framework, and it can ensure the flexibility, stability, scalability, and efficient handling ability for high-concurrency requests. The frontend is built using the Vue-based uni-app framework. As a cross-platform development solution, uni-app supports a single codebase to generate applications of both iOS and Android systems. The implementation interfaces of Haploid-Fertility are shown in Fig. 6.

## 4 Discussion

### 4.1 Different evaluation metrics for haploid fertility restoration

Maize haploid fertility restoration includes both female and male fertility restoration, and currently there is no unified standard for evaluating haploid fertility restoration. Strictly speaking, haploid fertility restoration refers to the ability of the tassel to produce viable pollen and achieve seed set via self-pollination. It is generally believed that the proportion of fertility restoration in haploid ears can exceed 90 ​%. However, there is larger variation in female fertility restoration, with most ears having only 1–5 seeds, while some materials can produce over a hundred seeds. Some studies use the average number of seeds per haploid ear to evaluate restoration ability, with the notion that any seed set on the ear is considered indicative of fertility restoration [2]. Furthermore, the size and length of haploid ears also vary greatly. Therefore, only using seed number to evaluate fertility restoration level is not appropriate. To this end, we propose the seed setting rate as a novel evaluation of the level for female fertility restoration, aligning with male fertility restoration standards. This metric can more intuitively and realistically reflect the degree of fertility restoration of haploid ear.

Currently, research on haploid fertility restoration predominantly focuses on haploid fertility. At the population level, the fertility restoration rate is usually defined as the proportion of haploid plants exhibiting anther emergence. At the individual level, there are more evaluation criteria for male, with most studies concentrating on the exposure of pollen and the amount of pollen released from the anthers. Some studies classify haploid male fertility into 4–5 levels based on the ratio of emergent anthers [55,56]. However, these classification criteria do not consider the quantity of pollen shed from the anthers of haploid tassel. Therefore, the fertility restoration evaluation indicator for pollen production in haploids is divided into three levels: (1) a small amount of fertile pollen; (2) a certain amount of fertile pollen; (3) nearly all pollen are fertile. To facilitate research on male fertility restoration from different genetic backgrounds, Wu et al. [33] classified fertility restoration into four genetic phenotypes: anther emergence rate, pollen exposure rate, anther emergence score, and pollen exposure score. Similar to disease resistance rating standards, the tassel anther emergence rate is a qualitative evaluation indicator, and some variation exists within each grade. Therefore, grading the phenotypes of haploid fertility restoration requires rich work experience of operators. Evaluating fertility restoration levels based on the pollen quantity of haploid tassels is currently challenging, and the definition of pollen quantity is prone to data bias. In summary, this study adopts anther emergence rate to evaluate haploid male fertility and it is practical and easier to implement.

### 4.2 Exploration of breeding efficiency and scalability in large-scale breeding scenarios

The Maize-IRNet and APP developed in this study are expected to improve SHGD efficiency, thereby shortening the breeding cycle and reducing costs. Normally, the cost of generating a single haploid plant (with chromosome doubling) is about ¥ 4 yuan. When the haploid doubling efficiency is 30 ​%, the total cost of producing 1000 DH lines is approximately ¥ 13 ​340 yuan. If the haploid doubling efficiency increases to 65 ​%, the cost can be reduced to about ¥ 6156 yuan, saving more than ¥ 7100 yuan compared with the original scheme. It suggests that a 1 ​% increase in doubling efficiency reduces production costs by about ¥ 205 yuan. In addition, Maize-IRNet facilitates the efficient collection of large-scale and accurate phenotype data of haploid fertility restoration in maize. This can further enhance the performance of genomic selection and promote the creation of new germplasm. Therefore, Maize-IRNet contributes to reducing breeding cycle and improving breeding efficiency, ultimately accelerating the development of new varieties.

In this study, we developed a maize haploid fertility restoration phenotyping and analysis platform based on the Maize-IRNet model. In the future, agriculture will develop towards digitization, scalability, and intelligence. Applying Maize-IRNet in large breeding programs requires addressing challenges of multi-scenario environments, automated hardware integration, and large-scale applications. In the future, we plan to integrate Maize-IRNet into field robots and low-altitude UAV platforms to improve the efficiency of large-scale field phenotyping of haploid fertility restoration. Due to the complex and diverse field environment, we will further train Maize-IRNet with large-scale and diverse datasets to enhance its robustness and adaptability. In addition, we will further integrate Maize-IRNet with germplasm evaluation, genomic selection, and breeding decision support systems, which will help achieve automation and intelligence throughout the entire process from phenotype collection to breeding decision-making.

### 4.3 Analysis of the structure and advantages of Maize-IRNet

The proposed Maize-IRNet demonstrates superior performance in the evaluation of maize haploid fertility, with the characteristics of multi-scale feature fusion architecture and global attention mechanism. Compared with the conventional deep learning methods, Maize-IRNet uses the composite structure of Inception-ResNet to effectively solve the coordination problem of local feature recognition and global structure perception in maize haploid fertility restoration images. Through the multi-branch convolutional network of Inception module, Maize-IRNet can accurately capture microscopic features (such as anther texture and seed color). Meanwhile, Maize-IRNet learns macroscopic features (ear size and tassel morphology) through its deep network by employing residual connections of ResNet. The global attention mechanism further optimizes the fusion of information from different scales by focusing on critical feature regions, thereby significantly enhancing the model performance in complex image recognition tasks. Base on the multi-scale feature fusion mechanism, Maize-IRNet achieves an accuracy of 84.2 ​% in the classification task of ear seed setting, with 2.7 ​% improvement over VGG networks. The result validates the crucial role of multi-scale feature fusion in complex task of maize haploid fertility.

The ablation experiments further validated the critical impact of each module on the performance. Removing the Inception-ResNet-B module results in the classification accuracy decreased by 3.7 ​% for fertility restoration, suggesting the necessity of cross-scale feature fusion. Through combining asymmetric convolution kernels with small convolution kernels, this module enables cross-scale perception of key biological indicators (such as anther density and number of seeds), thus providing essential discriminative features for subsequent classification tasks. To address the problem of information loss in conventional spatial feature dimensionality reduction, the Reduction module adopts a multi-branch compression architecture. Its parallel structure integrates multi-scale convolutions with max pooling operations, thereby reducing the network computational complexity while preserving feature integrity. This structure not only optimizes spatial feature dimensionality reduction but also enhances the applicability of grading model in field experiments.

Compared to the traditional attention mechanisms, the Global Attention Mechanism (GAM) captures the global dependencies of both channel and spatial information by performing element-wise multiplication between the channel and spatial weight matrices and the feature maps. This mechanism enables the model to focus on the key discriminative regions in the images (such as exposed anthers, seeds in the ear) by dynamically adjusting attention weights to enhance the recognition of target features. Experimental results indicate that the inclusion of GAM improves classification accuracy by 2.1 ​% under the same training conditions. The global dependency extraction function of the module could effectively increase attention to the target regions while mitigating interference of background and noise.

This study validates the effectiveness of a task-oriented network design architecture in the phenotype image analysis of maize haploid fertility restoration. By developing a prediction model based on the ratio of key features, we have preliminarily achieved a quantitative evaluation of fertility restoration levels in the images of maize haploid. The multi-scale feature fusion mechanism demonstrates excellent capability in extracting key features of seed distribution density and the level of anther emergence. The strategy of multi-branch parallel extraction and feature aggregation enables the model to adapt to fertility evaluation tasks with different architectures and classification criteria, thereby providing a novel solution for the phenotypic image analysis of maize.

### 4.4 Analysis of fuzzy level classification

In the evaluation of maize haploid fertility restoration, we classified the images into multiple levels based on anther emergence rate and seed setting rate (Table S1). However, the above classification criterion inevitably involves subjectivity and personal errors. For instance, the images of level 3 may be morphologically similar to those in adjacent levels (level 2 or level 4), and there are no strict boundaries for differentiation in practical breeding applications. It should be noted that level 0 represents either complete absence of seed (for ears) or no anther emergence (for tassels). Therefore, level 0 can reflect real phenotype features and cannot be classified as other levels.

To further investigate the impact of imprecise classification on prediction performance, we compared the accuracies of different methods in a fuzzy classification task. The accuracies of all methods for fuzzy classification are shown in Table S9. Experimental results indicate that the differences in prediction accuracy among all the methods were relatively small (with 1–3 misclassified images) in the fuzzy classification of ear seed setting. In the fuzzy classification results for the tassel anther emergence, both Maize-IRNet and VGG network achieve better fuzzy classification accuracies (91.9 ​%). Further analysis revealed that misclassifications were mainly concentrated on level 0 and level 1. In the case of misclassifying level 0 as level 1, Maize-IRNet has the fewest mispredictions (11 images), significantly outperforming the other methods. But there are more cases of misclassifying level 1 as level 0, indicating that Maize-IRNet tends to be conservative in its prediction of anther emergence rate, tends to predict a lower level (That is to say, underestimating the anther emergence rate). This result may be attributed to the following reasons: (1) GAM weights the feature maps through channel and spatial attention, and mainly focuses on key regions. In the cases of low anther emergence, the model may struggle to capture distinct anther features. Therefore, the relatively weak anther features might be further suppressed during feature extraction, making it more likely to misclassify images with low anther emergence as a lower level for the model. (2) The multi-scale feature fusion strategy of Maize-IRNet might be biased toward local high-contrast features when capturing the anther regions. But for the relatively sparse anther images, these weaker features lead the model to lean more towards lower anther emergence rate during decision-making. (3) The model may have learned a conservative estimation strategy because there are a large number of images without anthers exposed during the training process.

### 4.5 Application of automated phenotyping in digital agriculture

In the era of intelligent breeding, automated phenotypic data collection and analysis will play an increasingly important role. However, applying automated phenotyping in crop breeding requires careful consideration of data standardization, software-hardware coordination, data interpretability, and the precise real-time integration analysis of genotype and phenotype data. Compared with the manual phenotyping, automated pipelines involve standardized image acquisition, automated feature extraction, and software-hardware coordination. Data standardization and traceability are critical for automated phenotyping, including standardized processing of image resolution, analysis, management, and annotation. At the same time, the integration of Maize-IRNet into agricultural robots or low-altitude UAVs to achieve automated high-throughput phenotypic data collection is a necessary trend for future development. This requires designing and developing standardized, user-friendly interfaces that can be easily integrated with hardware for phenotypic data acquisition and analysis. Currently, researchers mainly use deep learning methods to analyze plant phenotype images. In the process of automated analysis, it is essential to visualize key image features to assist researchers in variety selection and uncovering underlying biological mechanisms. Finally, automated phenotyping systems must be thoroughly validated across diverse environmental conditions and genetic backgrounds. Moreover, real-time and automatic integration of the collected phenotypic data with genotype and transcriptome data will greatly accelerate both the elucidation of the genetic basis of haploid fertility restoration traits and the efficiency of crop improvement.

With the continuous development of artificial intelligence and integrated hardware-software technologies, digital agriculture and high-throughput phenotyping will play a key role in future digital farming. In this study, we developed an automated platform of Haploid-Fertility for haploid fertility restoration phenotyping, and it enables real-time field image acquisition, rapid data uploading, and accurate grading evaluation. In the future, integrating Maize-IRNet into agricultural robots or low-altitude UAVs will facilitate precise and high-throughput collection of large-scale phenotypic data on haploid fertility restoration. These data will contribute to the identification of related QTLs and the development of highly accurate genomic prediction models, thereby improving the efficiency of genome breeding and accelerate the development of new varieties.

### 4.6 Shortcomings and prospects

Although this study demonstrates that Maize-IRNet outperforming the classical deep learning methods in terms of grading accuracy and computational efficiency of maize haploid fertility restoration traits, there are also some limitations. Due to the factors of image quality (e.g., lighting variations) and sample size, the prediction accuracy for certain fertility restoration levels is relatively low. We plan to use contrastive learning to optimize Maize-IRNet and further improve prediction accuracy. In addition, we will collect diverse phenotypic image datasets under complex field backgrounds, including lighting changes and various occlusions, to enhance the robustness and generalization ability of Maize-IRNet. Moreover, more precise three-dimensional (3D) structural information can compensate for the limitations of 2D images in feature representation, enabling higher accuracy evaluation of haploid fertility restoration phenotypes. In the future, we plan to introduce automated 3D laser scanning devices to achieve high-throughput acquisition of 3D structural information of haploid tassels and ears. Overall, this study enables high-accuracy evaluation of haploid fertility restoration phenotypes in maize. It also establishes a systematic and scalable framework for automated phenotypic data collection and analysis in future double haploid breeding application.

In addition, this study proposes a grading evaluation model (Maize-IRNet) for haploid tassel anther emergence and ear seed setting, and develops a mobile application. The platform enables real-time field image acquisition, rapid data uploading, and grading evaluation. In the future, integrating Maize-IRNet into agricultural robots or low-altitude UAVs will provide precise and high-throughput phenotype data for studying the SHGD mechanism. This will greatly accelerate QTL mapping of haploid fertility restoration traits in maize, thereby facilitating the cloning of SHGD genes. Furthermore, haploid genome doubling remains a major bottleneck limiting the large-scale application of DH technology. Currently, chemical doubling methods are still the main approach in production. The developed APP of Maize-IRNet model can provide accurate and efficient phenotype data for individual plants. This will support the optimization of chemical doubling strategies and the selection of optimal doubling combinations, thereby accelerating the adoption of DH technology in practical breeding programs.

## 5 Conclusions

This study combines deep learning with high-throughput field phenotyping technology to develop a grading model and mobile application for maize haploid fertility restoration. Firstly, we constructed a dataset containing 1897 high-resolution haploid ear images with different seed setting rates and 6443 tassel images with varying anther emergence rates. This dataset effectively alleviates the scarcity of phenotype images of maize haploid fertility restoration. Second, we proposed Maize-IRNet, a grading evaluation model for haploid fertility restoration traits based on Inception-ResNet. Compared with the classical methods of VGG, ResNet, ViT, MLP-Mixer, and gMLP, Maize-IRNet achieves higher prediction accuracy. Finally, we developed an Android mobile application integrating the Maize-IRNet algorithm. The application enables real-time field image acquisition, uploading, analysis, and grading. This study provides a practical technical solution for efficient acquisition and analysis of maize haploid fertility restoration phenotypes, which can be directly applied in field breeding practices.

## Author contributions

Y.Q. and J.L. propose the concept and managed the project. Y.W. and Z.Y. contributed the methodology and experimental design. Y.W., Y.Q. and J.L. contributed to the writing. W.S. and Y.Q. provided dataset. K.J. and F.Z. developed the mobile application of Haploid-Fertility. L.J. contributed to funding. J.D., Y.X., Z.Z., J.Y., J.L. and Y.Q. supervised and edited the manuscript. All authors discussed the results, provided feedback, and contributed to the writing of the manuscript.

## Funding

This work was supported by the 10.13039/501100012166National Key Research and Development Program of China [2022YFD1201504], National Natural Science Foundation of China [32572406], Major Program(JD) of Hubei Province [2025BEA003], 10.13039/501100003819Hubei Provincial Natural Science Foundation [2023AFB832], Guizhou Provincial Basic Research Program (Natural Science) [MS[2025]096], Major Project of Hubei Hongshan Laboratory [2022HSZD031].

## Data availability

The maize haploid fertility image dataset collected by smartphones is available at https://github.com/wyzwyz666/maize-haploid-fertility/blob/main/dataset. Application and source code availability: Maize-Fertility APP: https://github.com/wyzwyz666/maize-haploid-fertility/releases/download/v1.1.0/Haploid-Fertility.apk. The source code: https://github.com/wyzwyz666/maize-haploid-fertility/blob/main/sourcecode.

## Competing interests

The authors declare that they have no competing interests.
