# Supplementary material for: Grading evaluation of haploid fertility restoration traits based on inception-ResNet in maize
Source: Plant Phenomics. 2025 Nov 7;7(4):100140. doi: 10.1016/j.plaphe.2025.100140 (PMC13109345; doi:10.1016/j.plaphe.2025.100140)
Supplement: Multimedia component 1 [file mmc1.docx]

**Supplemental Information**

# **Supplemental Tables**

**Table S1.** Evaluation criteria of fertility restoration in maize haploid plants.

| Dataset | Level | Details |
| --- | --- | --- |
| Ear seed setting rate | level 0 | No seed setting in ears: 0% |
|  | level 1 | Seed setting rates: 1%-5% |
|  | level 2 | Seed setting rates: 6%-20% |
|  | level 3 | Seed setting rates: 21%-50% |
|  | level 4 | Seed setting rates: 51%-75% |
|  | level 5 | Seed setting rates: >75% |
| Tassel anther emergence rate | level 0 | No anther emergence in tassels: 0% |
|  | level 1 | Anther emergence rates: 1%-5% |
|  | level 2 | Anther emergence rates: 6%-20% |
|  | level 3 | Anther emergence rates: 21%-50% |
|  | level 4 | Anther emergence rates: 51%-75% |
|  | level 5 | Anther emergence rates: >75% |

**Table S2.** Ablation experiment results of the modules in Maize-IRNet on tassel anther emergence rate.

| Name | Remove Module | Accuracy | Recall rate | Precision | F1-Score |
| --- | --- | --- | --- | --- | --- |
| Backbone-1 | Inception-resnet-A | 82.0 | 72.9 | 73.0 | 72.9 |
| Backbone-2 | Reduction-A | 80.9 | 68.8 | 68.6 | 68.7 |
| Backbone-3 | Inception-resnet-B | 81.5 | 69.2 | 71.6 | 70.4 |
| Backbone-4 | Reduction-B | 72.1 | 52.6 | 51.7 | 52.1 |
| **Backbone** | **-** | **82.5** | **71.6** | **71.9** | **71.7** |

**Table S3.** Comparison performance of integrating four attention mechanisms in the backbone network on tassel anther emergence rate.

| Methods | Accuracy | Recall rate | Precision | F1-Score |
| --- | --- | --- | --- | --- |
| Backbone | 82.5 | 71.6 | 71.9 | 71.7 |
| Backbone+CBAM | 82.2 | 70.4 | 72.3 | 71.3 |
| Backbone+SE | 81.7 | 71.1 | 72.2 | 71.6 |
| Backbone+EMA | 81.1 | 70.1 | 71.1 | 70.6 |
| **Backbone+GAM** | **84.0** | **75.3** | **74.3** | **74.8** |

**Table S4.** The classification results of different initialization methods of Maize-IRNet for haploid ear seed setting rate.

| Initialization methods | Accuracy | Recall rate | Precision | F1-Score |
| --- | --- | --- | --- | --- |
| Normal | 83.2 | 83 | 82.7 | 82.8 |
| Xavier | 83.6 | 83 | 83.5 | 83.2 |
| Orthogonal | 83.1 | 82.5 | 82.5 | 82.5 |
| **He** | **84.2** | **83.7** | **83.8** | **83.7** |

**Table S5.** Samples size of tassel anther emergence and ear seed setting.

| Level | Anther emergence | Seed setting |
| --- | --- | --- |
| 0 | 3,279 | 233 |
| 1 | 1,145 | 498 |
| 2 | 500 | 321 |
| 3 | 487 | 315 |
| 4 | 422 | 210 |
| 5 | 610 | 320 |

**Table S6.** Experimental results of Maize-IRNet of ten-fold cross-validation.

| Dataset | Accuracy | Recall rate | Precision | F1-Score |
| --- | --- | --- | --- | --- |
| Ear seed setting rate | 82.9 ± 0.9 | 82.1 ± 1.4 | 82.1 ± 1.2 | 82.1 ± 1.2 |
| Tassel anther emergence rate | 83.0 ± 1.8 | 72.1 ± 3.1 | 73.7 ± 2.7 | 72.9 ± 3.0 |

**Table S7.** Comparison results of accuracy, GPU memory, and runtime of Maize-IRNet at different image resolutions.

| Dataset | Methods | Accuracy | GPU memory (GB) | train time (s) | test time (s) |
| --- | --- | --- | --- | --- | --- |
| Ear seed setting rate | 2048 batch8 | 84.2 | 31.2 | 117 | 59 |
|  | 299 batch8 | 83.2 | 2.0 | 24 | 15 |
|  | **299 batch16** | **84.2** | **2.9** | **19** | **15** |
| Tassel anther emergence rate | 2048 batch8 | 82.1 | 31.2 | 444 | 246 |
|  | 299 batch8 | 82.3 | 2.0 | 60 | 45 |
|  | **299 batch16** | **84.0** | **2.9** | **46** | **45** |

**Table S8.** The running time and parameter size of different models.

| Model | Test time(s) | Total Parameters (MB) |
| --- | --- | --- |
| VGG11_bn | 0.00791 | 491.32 |
| ResNet50 | 0.00527 | 89.72 |
| ResNet101 | 0.00861 | 162.17 |
| ViT-Base-16 | 0.02616 | 326.73 |
| gMLP | 0.00158 | 11.33 |
| MLP-Mixer | 0.00515 | 80 |
| **Maize-IRNet** | **0.00663** | **137.15** |

**Table S9.** Fuzzy classification results of seed setting rate and anther emergence rate.

| Type | Methods | Accuracy |
| --- | --- | --- |
| Seed setting rate | VGG11_bn | 99.4 |
|  | ResNet50 | 98.9 |
|  | ResNet101 | 98.9 |
|  | Maize-IRNet | 98.4 |
| Anther emergence rate | VGG11_bn | 91.9 |
|  | ResNet50 | 89.7 |
|  | ResNet101 | 89.4 |
|  | Maize-IRNet | 91.9 |

# **Supplemental Figures**


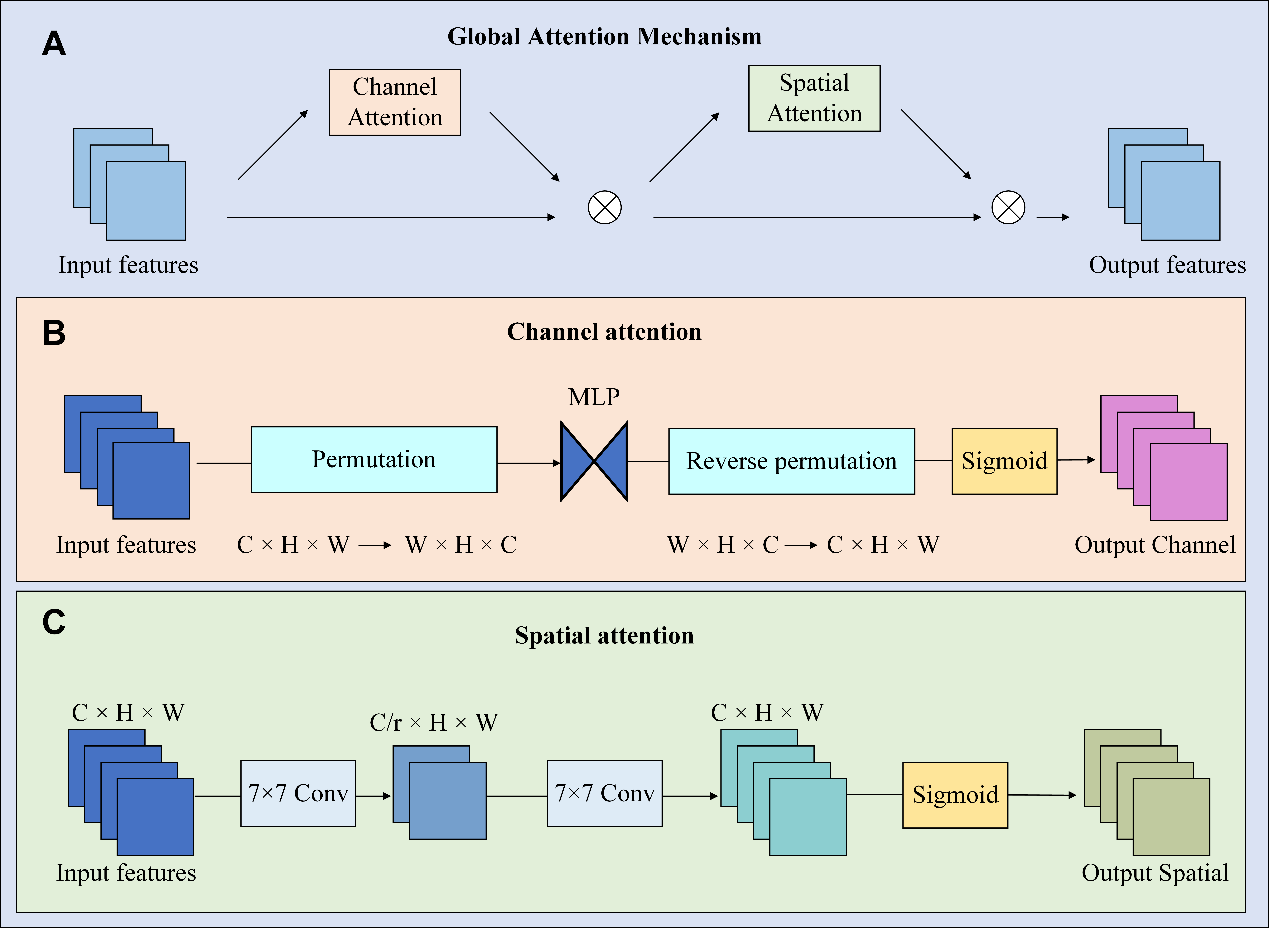


Fig. S1. Architecture of the global attention mechanism (GAM). (A) The overall architecture of GAM. (B) The architecture of the Channel attention. (C) The architecture of the Spatial attention.


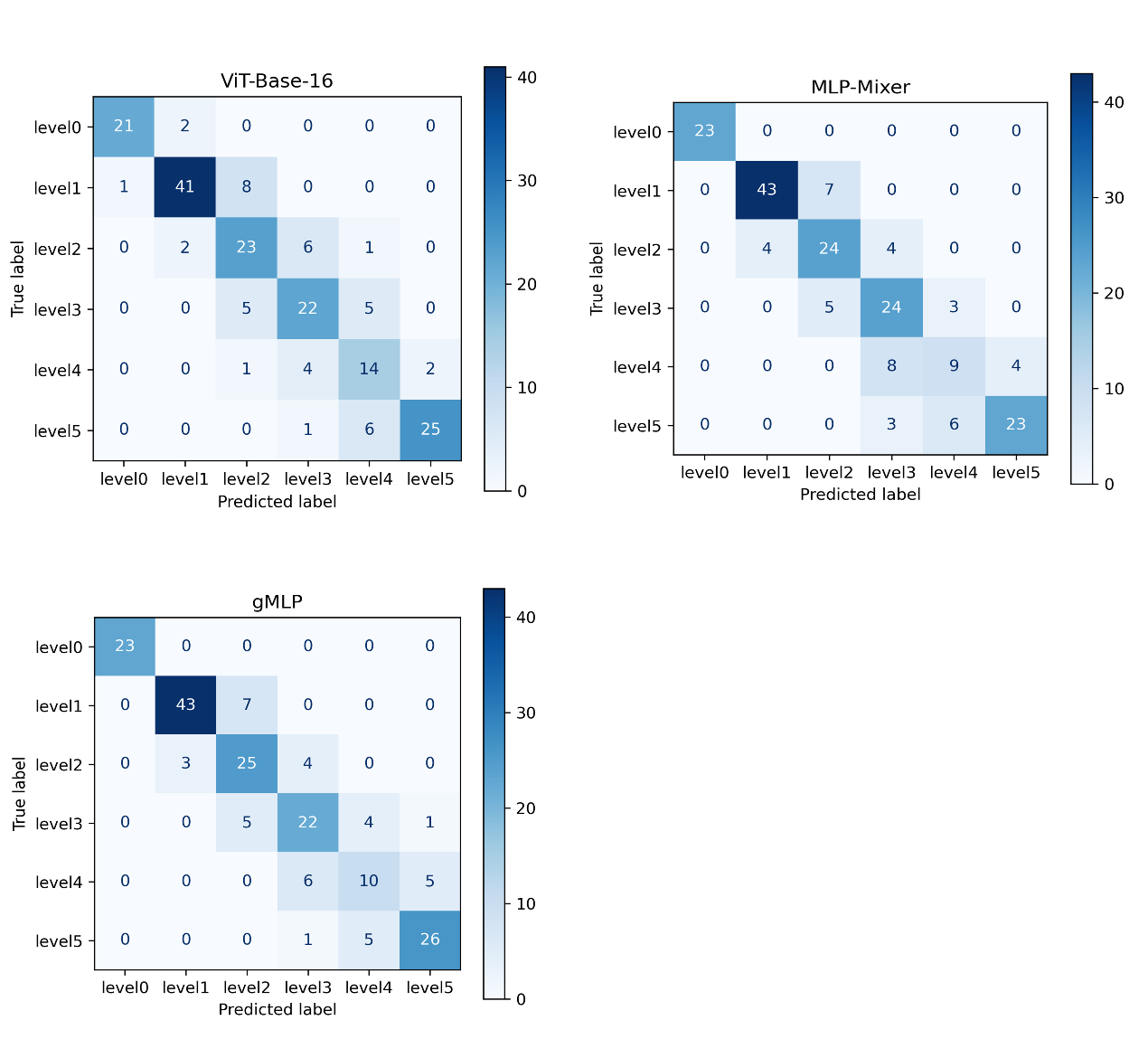


Fig. S2. The grading confusion matrix for ear seed setting rate of ViT-Base-16, MLP-Mixer, gMLP.


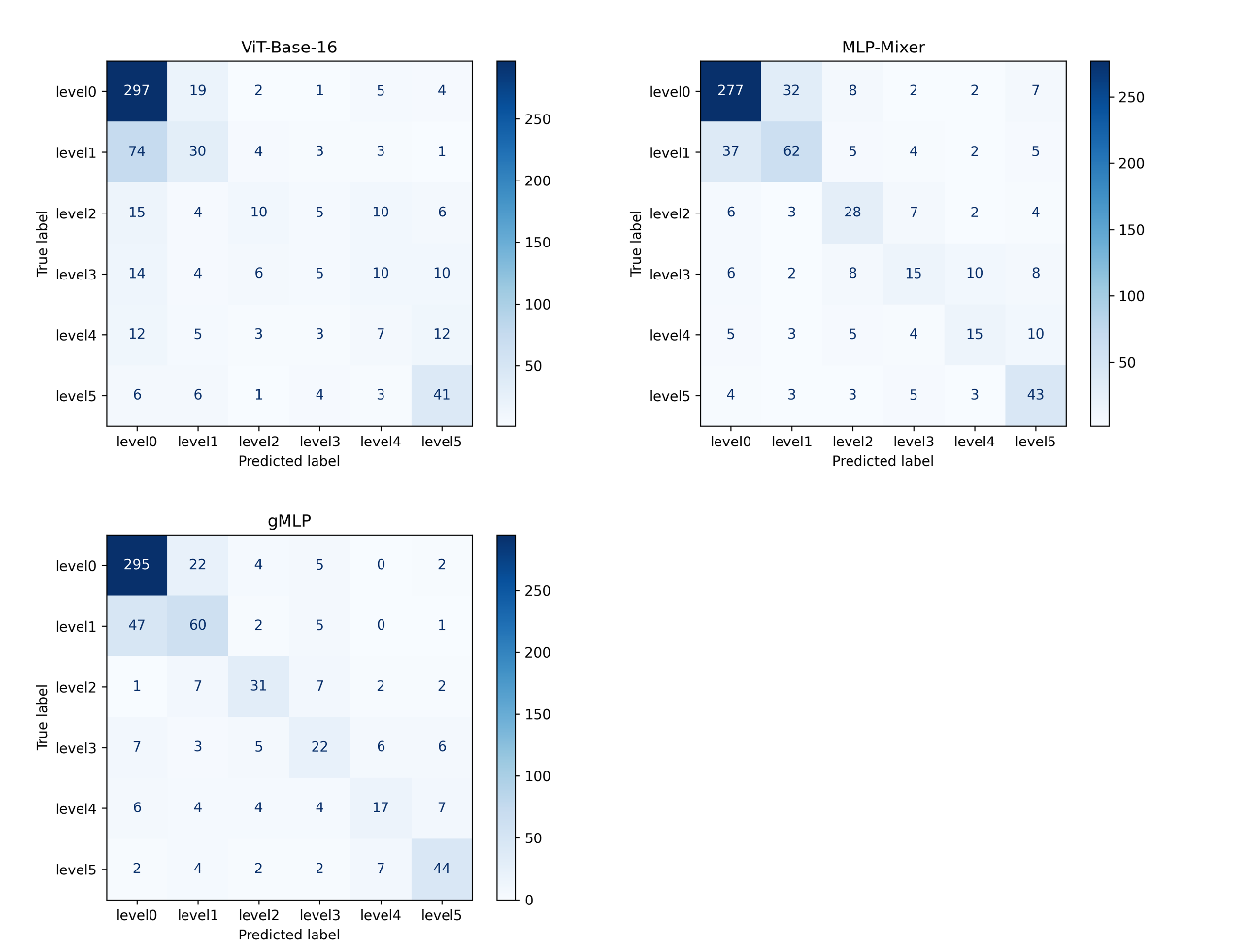


Fig. S3. The grading confusion matrix for tassel anther emergence rate of ViT-Base-16, MLP-Mixer, gMLP.


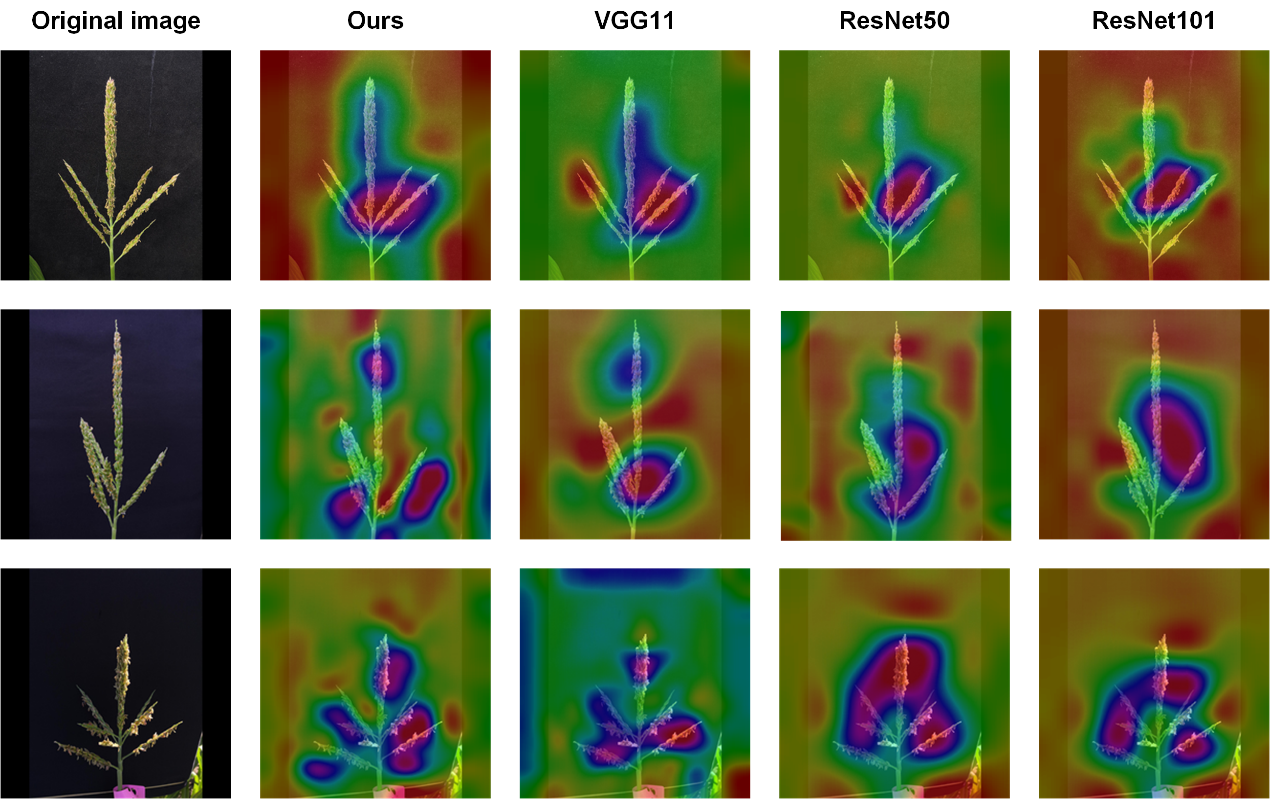


Fig. S4. Feature attention heatmap of VGG11_bn, ResNet50, ResNet101 and Maize-IRNet of haploid tassel anther emergence rate grading.
